# Variability and Diversity of Nasopharyngeal Microbiota in Children: A Metagenomic Analysis

**DOI:** 10.1371/journal.pone.0017035

**Published:** 2011-02-28

**Authors:** Debby Bogaert, Bart Keijser, Susan Huse, John Rossen, Reinier Veenhoven, Elske van Gils, Jacob Bruin, Roy Montijn, Marc Bonten, Elisabeth Sanders

**Affiliations:** 1 Department of Paediatric Infectious Diseases and Immunology, University Medical Center Utrecht-Wilhelmina Children's Hospital, Utrecht, The Netherlands; 2 Business Unit Food and Biotechnology Innovations, Microbial Genomics Group, TNO Quality of Life, Zeist, The Netherlands; 3 Marine Biological Laboratory, Josephine Bay Paul Center for Comparative Molecular Biology and Evolution, Woods Hole, Massachusetts, United States of America; 4 Laboratory of Medical Microbiology and Immunology, St. Elisabeth Hospital, Tilburg, The Netherlands; 5 Linnaeus Institute, Spaarne Hospital, Hoofddorp, The Netherlands; 6 Regional Laboratory of Public Health, Haarlem, The Netherlands; 7 Department of Medical Microbiology, University Medical Center Utrecht, Utrecht, The Netherlands; 8 Julius Center of Health Sciences and Primary Care, University Medical Center Utrecht, Utrecht, The Netherlands; University of Liverpool, United Kingdom

## Abstract

The nasopharynx is the ecological niche for many commensal bacteria and for potential respiratory or invasive pathogens like *Streptococcus pneumoniae*, *Haemophilus influenzae*, and *Neisseria meningitidis*. Disturbance of a balanced nasopharyngeal (NP) microbiome might be involved in the onset of symptomatic infections with these pathogens, which occurs primarily in fall and winter. It is unknown whether seasonal infection patterns are associated with concomitant changes in NP microbiota. As young children are generally prone to respiratory and invasive infections, we characterized the NP microbiota of 96 healthy children by barcoded pyrosequencing of the V5–V6 hypervariable region of the 16S-rRNA gene, and compared microbiota composition between children sampled in winter/fall with children sampled in spring. The approximately 1000000 sequences generated represented 13 taxonomic phyla and approximately 250 species-level phyla types (OTUs). The 5 most predominant phyla were Proteobacteria (64%), Firmicutes (21%), Bacteroidetes (11%), Actinobacteria (3%) and Fusobacteria (1,4%) with Moraxella, Haemophilus, Streptococcus, Flavobacteria, Dolosigranulum, Corynebacterium and Neisseria as predominant genera. The inter-individual variability was that high that on OTU level a core microbiome could not be defined. Microbiota profiles varied strongly with season, with in fall/winter a predominance of Proteobacteria (relative abundance (% of all sequences): 75% versus 51% in spring) and Fusobacteria (absolute abundance (% of children): 14% versus 2% in spring), and in spring a predominance of Bacteroidetes (relative abundance: 19% versus 3% in fall/winter, absolute abundance: 91% versus 54% in fall/winter), and Firmicutes. The latter increase is mainly due to (Brevi)bacillus and Lactobacillus species (absolute abundance: 96% versus 10% in fall/winter) which are like Bacteroidetes species generally related to healthy ecosystems. The observed seasonal effects could not be attributed to recent antibiotics or viral co-infection.

The NP microbiota of young children is highly diverse and appears different between seasons. These differences seem independent of antibiotic use or viral co-infection.

## Introduction

According to the WHO, respiratory tract infections are still among the leading causes of death in children and adults worldwide [1]. The most common pathogens like *Streptococcus pneumoniae, Haemophilus influenzae, Neisseria meningitidis* and *Staphylococcus aureus* are normal and transient residents of the nasopharyngeal (NP) niche, where they are embedded in a complex microbiota of generally presumed harmless commensals. The human microbiome in general is assumed beneficial to the host due to stimulation and maturation of immune systems, promotion of mucosal structure and function and providing actual ‘colonization resistance’ against pathogen invasion [2]. Although colonization by the “potential pathogens” of the NP microbiome is mainly asymptomatic, progression towards upper respiratory tract infections, pneumonia or even sepsis and meningitis may occur [3], [4]. The exact mechanisms by which this occurs remain largely unknown, although an imbalance in the composition of microbiota, for example by acquisition of new pathogens, viral co-infection or other host or environmental factors have been suggested [5]–[8]. In addition, clear correlations between invasive attack rates and season are observed for many of the potential pathogens of the upper respiratory tract [9], [10], a phenomenon that cannot be fully explained by concomitant changes in colonization rates of the individual pathogenic bacteria [11], [12]. This suggests that local containment of the colonizing pathogenic bacteria by the host and/or the surrounding ecosystem is of major importance in prevention of disease progression. Despite an abundance of data on incidence, prevalence and density of potential pathogens in NP microbiota of children and adults, the detailed composition of the NP microbial community, both during health and disease have not been studied. We, therefore, performed a meta-genomic study on the detailed composition of and variability in NP microbiota in young children sampled during different seasons.

## Results and Discussion

**Figure 1:**
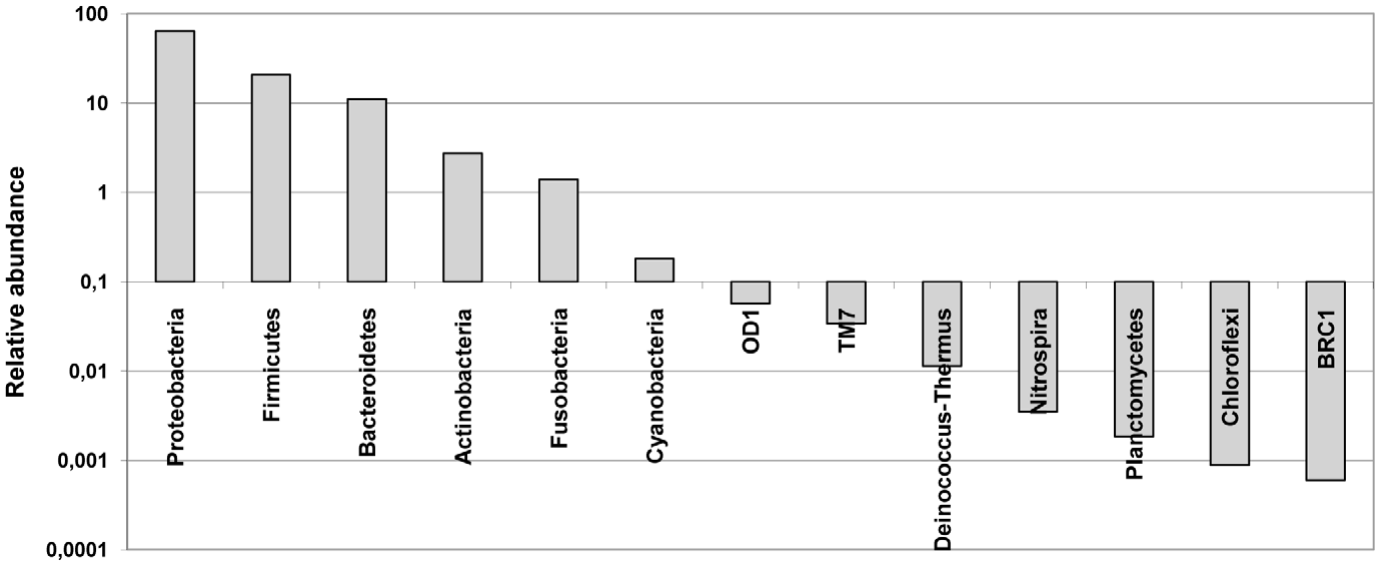
Relative abundance of all bacterial phyla found in the NP microbiota of 96 infants 18 months of age. A cut-off of 0.1% is used for visual differentiation between predominant and less dominant phyla.

We studied the NP microbiota composition of 96 healthy 18-months old children. Their characteristics are depicted in Table S1. Being aware of the current discussions on the artefacts that may be introduced by pyrosequencing [13], [14], we applied a stringent protocol for filtering and clustering of sequences. The approx. 1 100 000 generated sequences (on average 11000 sequences per sample) yielded about 92 000 unique sequences, representing 13 taxonomic phyla and 243 species-level phyla types (OTUs). The data were normalized for equal numbers of reads per sample. The 5 most predominant phyla were Proteobacteria (64%), Firmicutes (21%), Bacteroidetes (11%), Actinobacteria (3%) and Fusobacteria (1.4%) (Figure 1). In addition, we found representatives of Cyanobacteria, probably reflecting plant chloroplasts obtained through inhalation. Sporadically and/or in low abundance we found sequences for the candidate divisions OD1, TM7 and BRC1 and the phyla Deinococcus-Thermus, Nitrospira, Planctomycetes and Chloroflexi. On a lower taxonomic level, the most prevalent genera were Moraxella (40%), Haemophilus (20%), Streptococcus (12%), and Flavobacterium (10%). Other fairly common genera were Dolosigranulum (5%), Corynebacterium (2%), Neisseria (2%) and Fusobacterium (1%). The 30 most common OTUs representing almost 98% of all reads, and their relative and absolute presence are shown in Table 1 (For the complete list of OTUs; see Table S2). Although the top 6 predominant phyla are identical to those of neighbouring microbiota, the composition, i.e. relative contribution of each phyla to those microbiota seems fairly different. In the oral cavity, microbiota are dominated by Firmicutes followed by Proteobacteria and Bacteroidetes (overall 50% Gram-positive bacteria), whereas the microbiome of the nostril contains more than 80% gram-positive bacteria, mostly Actinobacteria and Firmicutes [15]. These data, therefore, suggest different dynamics (i.e., different biological equilibria) in the NP microbiome.

**Table 1:** Thirty most common OTUs or ‘species-level’ phylotypes (ranked by predominance, i.e. absolute presence among the approx. 1 100 000 reads).

	Phylum	Class	Order	Family	Genus	Species	OTU level classified	Overall presence (% of reads)	Relative presence (n = )	Relative presence (>0.1% of reads) (n = )	Core microbiome
1	Proteobacteria	Gammaproteobacteria	Pseudomonadales	Moraxellaceae	Moraxella	NA	genus	38.11	95	91	All
2	Proteobacteria	Gammaproteobacteria	Pasteurellales	Pasteurellaceae	Haemophilus	influenzae	species	19.16	84	61	All
3	Firmicutes	Bacilli	Lactobacillales	Streptococcaceae	Streptococcus	NA	genus	12.98	96	88	All
4	Bacteroidetes	Flavobacteria	Flavobacteriales	Flavobacteriaceae	Flavobacterium	NA	genus	10.07	80	47	Spring
5	Firmicutes	Bacilli	Lactobacillales	Carnobacteriaceae	Dolosigranulum	NA	genus	4.80	86	75	All
6	Proteobacteria	Gammaproteobacteria	Pseudomonadales	Moraxellaceae	Moraxella	NA	genus	2.22	39	28	
7	Actinobacteria	Actinobacteria	Actinomycetales	Corynebacteriaceae	Corynebacterium	propinquum	species	1.65	80	57	All
8	Proteobacteria	Betaproteobacteria	Neisseriales	Neisseriaceae	Neisseria	meningitidis	species	1.19	62	22	
9	Fusobacteria	Fusobacteria	Fusobacteriales	Fusobacteriaceae	Fusobacterium	necrophorum	species	0.96	8	4	
10	Proteobacteria	Gammaproteobacteria	Pasteurellales	Pasteurellaceae	Haemophilus	influenzae	species	0.77	16	10	
11	Proteobacteria	Betaproteobacteria	Neisseriales	Neisseriaceae	Neisseria	polysaccharea	species	0.65	16	7	
12	Firmicutes	Clostridia	Clostridiales	Peptostreptococcaceae	Helcococcus	NA	genus	0.57	31	23	
13	Firmicutes	NA	NA	NA	NA	NA	phylum	0.57	49	23	
14	Actinobacteria	Actinobacteria	Actinomycetales	Dermabacteraceae	Brachybacterium	NA	genus	0.56	16	11	
15	Fusobacteria	Fusobacteria	Fusobacteriales	Fusobacteriaceae	Fusobacterium	NA	genus	0.40	21	2	
16	Proteobacteria	Gammaproteobacteria	Pseudomonadales	Moraxellaceae	Enhydrobacter	NA	genus	0.37	95	73	All
17	Proteobacteria	Gammaproteobacteria	Pasteurellales	Pasteurellaceae	Haemophilus	NA	genus	0.34	14	8	
18	Bacteroidetes	Bacteroidia	Bacteroidales	Porphyromonadaceae	Porphyromonas	catoniae	species	0.27	29	9	
19	Firmicutes	Bacilli	Lactobacillales	Lactobacillaceae	Lactobacillus	NA	genus	0.24	17	9	
20	Bacteroidetes	Bacteroidia	Bacteroidales	Porphyromonadaceae	Porphyromonas	catoniae	species	0.21	23	9	
21	Firmicutes	Clostridia	Clostridiales	Peptostreptococcaceae	Parvimonas	NA	genus	0.19	8	5	
22	Cyanobacteria	NA	NA	NA	NA	NA	phylum	0.18	83	46	Fall/Winter
23	Firmicutes	Bacilli	Lactobacillales	Streptococcaceae	Streptococcus	NA	genus	0.17	25	5	
24	Firmicutes	Bacilli	Bacillales	Paenibacillaceae	Brevibacillus	brevis	species	0.16	43	34	Spring
25	Bacteroidetes	Bacteroidia	Bacteroidales	Prevotellaceae	Prevotella	shahii	species	0.15	1	1	
26	Firmicutes	Bacilli	Bacillales	Bacillaceae	Bacillus	NA	genus	0.14	42	33	Spring
27	Actinobacteria	Actinobacteria	Actinomycetales	Propionibacteriaceae	Propionibacterium	NA	genus	0.13	90	43	Fall/Winter
28	Firmicutes	Bacilli	Bacillales	Staphylococcaceae	Staphylococcus	NA	genus	0.12	80	31	
29	Firmicutes	Clostridia	Clostridiales	Lachnospiraceae	NA	NA	family	0.12	15	7	
30	Proteobacteria	Betaproteobacteria	Burkholderiales	Comamonadaceae	Acidovorax	NA	genus	0.11	86	31	

Nr. of samples (of total of 96 samples) containing each OTU in >0% or >0.1% of the reads is stated. Core microbiome: OTUs found in >50% of the samples in >0.1% of reads per sample (All: OTU found in >50% of samples; Spring and Fall/Winter: OTU found in >50% of samples obtained in spring or fall/winter, respectively). NA: not assigned.

There was a high inter-individual variability in the composition of the microbiota up to phyla level, and in the relative abundance of the individual bacterial inhabitants (Table 1). This resulted in a limited core microbiome (as representing >0.1% of sequenes and being present in all 96 children) of specific phyla only, namely Proteobacteria and Firmicutes, however no single OTU was found universally. Because of the observed high inter-individual variation, we applied a less strict definition of core microbiome, i.e. OTUs present in more than 50% of all samples and representing >0.1% of the sequences. With this definition we observed a core microbiome of Moraxella, *Haemophilus influenzae*, Enhydrobacter (Proteobacteria), Streptococcus, Dolosigranulum (Firmicutes), and Corynebacterium (Actinobacteria) (Table 1).

**Figure 2:**
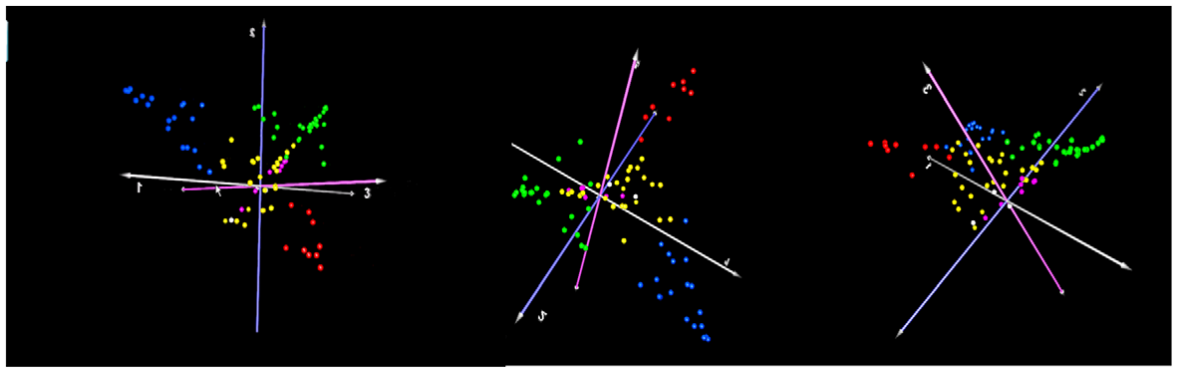
Principal component analysis of the individual NP communities. We observed three individual clusters/axes surrounding a centre of profiles, with in green individual profiles depicting predominantly (>50% of sequences) Moraxella OTU 1, in blue microbiota profiles depicting predominantly *H. influenzae* OTU 2, and in red microbiota profiles depicting predominantly Streptococcus OTU 3. Mixed phyla profiles (no single OTU is representing >50% of sequences) cluster in the centre of this PCA plot.

Principal component analysis identified 3 distinct clusters of microbiota profiles correlating strongly with a predominance (>50% of sequences per sample) of single OTU's, i.e. Moraxella (OTU 1), *Haemophilus influenzae* (OTU 2), and Streptococci (OTU 3), respectively, connected by a group of community profiles representing mixed microbiota (Figure 2). Additionally, we observed transition zones for microbiota profiles between the Haemophilus- and Moraxella-dominated clusters, but not between Haemophilus- or Moraxella-dominated clusters and the Streptococci-dominated cluster, which might implicate potential interactions between microbiota profiles.

**Figure 3:**
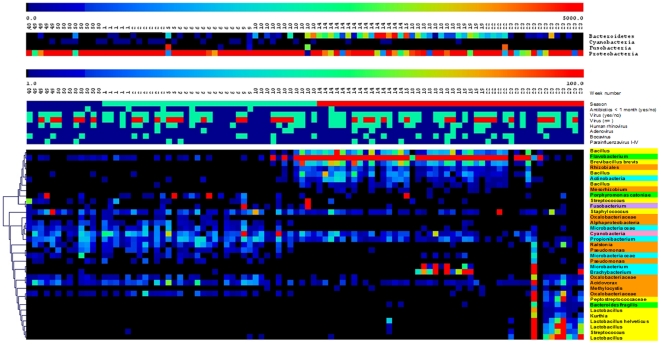
Seasonal differences between microbiota profiles of 50 children sampled in fall-winter and 46 children in spring. The samples are marked by the week number they were obtained (week 48 until week 23). In figure 3a the phyla showing significant association with season of sampling by SAM analysis are depicted. In figure 3b the OTUs showing significant association with season are depicted. The samples are marked by season (blue; fall, green; winter, red; spring), antibiotic use (<1 month: green), presence of viruses (positive: green), presence of multiple viruses (green: 1 virus, red: ≥ 2 viruses), Presence of Human rhinovirus, Adenovirus, Bocavirus, and Para-influenzavirus I-IV (positive: green). Groups of OTUs belonging to specific phyla are depicted with separate colours; Yellow: Firmicutes, Orange: Proteobacteria, Green: Bacteroidetes, Blue: Actinobacteria, Pink: Cyanobacteria.

Since respiratory and invasive infections are associated with fall/winter season, we analysed our samples concordantly. When distinguished by the time of sampling (fall/winter versus spring) groups of children did not differ significantly in demographics or life style characteristics, infectious symptoms, medical history or environmental parameters (Table S1), reducing the likelihood of internal confounders as a cause of potential seasonal correlations with microbiota profiles. However, with respect to microbiota profiles, we observed marked differences between samples obtained in fall/winter versus samples from spring (Table 2). In samples obtained in late fall and winter, we observed a predominance of Proteobacteria (relative abundance (% of all sequences): 75% versus 51% in spring), Fusobacteria (absolute abundance (% of children): 14% versus 2% in spring), and Cyanobacteria (absolute abundance: 64% versus 30% in spring; relative abundance: 0.27% versus 0.09% in spring) were significantly more abundant compared to spring, whereas Bacteroidetes were more frequently present in samples obtained in spring (relative abundance: 19% versus 3% in fall/winter, absolute abundance: 91% versus 54% in fall/winter) (Figure 3a). On OTU level we observed amongst others more Bacillus, Brevibacillus and Lactobacillus species, and Flavobacterium and *B. fragilis* (both Bacteroidetes) in samples from spring compared to fall/winter. In addition we found less α-Proteobacteria, Oxalobacteriaceae, Microbacteriaceae, Ralstonia, Pseudomonas and Acidovorax (all Proteobacteria), Cyanobacteria, and *Porphyromonas catoniae* (Bacteroidetes) in samples from spring compared to fall/winter (Figure 3b). When re-evaluating the core microbiome per individual season (i.e. OTUs present in more than 50% of samples of a certain season), we observed an additional core of Proprionibacterium and Cyanobacteria for fall/winter and an additional core of Flavobacteria, Brevibacillus and Bacillus (almost exclusively) for spring. The latter groups of bacteria, i.e. Bacteroidetes and (Brevi)bacillus and Lactobacillus species, are generally related to protection against overgrowth of pathogenic species due to the production of bacteriocins and other inhibitory substances [7], [16]. In other microbiota like the gastrointestinal and vaginal tract they are highly related to maintenance of a balanced microbiome as well [17]–[19]. Since infections with respiratory pathogens, especially pneumonia, are strongly related to fall and winter season [9], [10], the presence and abundance of these bacteria in respiratory microbiota in spring might therefore suggest in general a more balanced respiratory microbiome in this specific season as well protecting against onset of respiratory or invasive infections.

**Table 2:** Relative abundance of individual phyla is depicted per season.

		Fall/Winter					Spring				MWU
Phyla	Mean (%)	SD (%)	Min (%)	Max (%)	absolute nr	Mean (%)	SD (%)	Min (%)	Max (%)	absolute nr	p-value
Proteobacteria	**75.39**	23.81	1.91	98.41	50	**51.13**	23.77	8.09	98.89	46	<0.001
Firmicutes	**17.22**	16.58	0.30	67.64	50	**24.76**	25.22	0.78	91.18	46	0.097
Bacteroidetes	**3.39**	8.99	0.00	43.45	27	**19.31**	16.62	0.00	55.42	42	<0.001
Actinobacteria	**1.88**	3.36	0.05	22.69	49	**3.67**	5.91	0.02	29.40	42	NS
Fusobacteria	**1.75**	8.14	0.00	48.00	7	**0.98**	6.62	0.00	44.88	1	NS
Cyanobacteria	**0.27**	0.44	0.00	2.54	32	**0.09**	0.13	0.00	0.74	14	0.029
OD1	**0.067**	0.086	0.000	0.38	12	**0.046**	0.054	0.000	0.19	9	NS
TM7	**0.045**	0.305	0.000	2.15	1	**0.023**	0.151	0.000	1.02	1	NS
Deinococcus-Thermus	**0.015**	0.030	0.000	0.14	3	**0.008**	0.014	0.000	0.06	0	NS
Nitrospira	**0.005**	0.012	0.000	0.05	0	**0.001**	0.005	0.000	0.02	0	0.103
Planctomycetes	**0.001**	0.008	0.000	0.06	0	**0.002**	0.013	0.000	0.08	0	NS
Chloroflexi	**0.001**	0.005	0.000	0.03	0	**0.001**	0.005	0.000	0.04	0	NS
BRC1	**0.000**	0.000	0.000	0.00	0	**0.001**	0.009	0.000	0.06	0	NS

Mean, SD, and Range of each phyla per group of samples are depicted for the samples obtained in fall/winter (n = 50) versus samples obtained in spring (n = 46).

We tested all samples for the presence of respiratory viruses by q-PCR methods and detected one or more viruses in 67% of samples (Table S3). We found no evidence for associations between the observed seasonal shift in microbiota and the overall presence of respiratory viruses, nor for any of the individual viruses when tested by SAM analysis (Figure 3b). Although these data do not exclude an effect of respiratory viruses on microbiota composition, they do suggest other season-related factors like environmental factors (temperature, humidity, smoke exposure, crowding), or nutrient- or vitamine-related effects, or a combination of factors might be important for the observed shifts in microbiota profiles [3], [16], [20]. Interestingly, day-care attendance or smoke exposure could not be related to the observed shifts in microbiota, although the latter was encountered very rarely. This further underlines that different or more complex effect may be responsible for the observed phenomenon. In addition, no association was observed between seasonal changes in microbiota and recent antibiotic use, which was rather limited in this population (Table 1, Figure 3b). Because of the explicit correlation between season and microbiota profiles, correlations between other environmental determinants and microbiota could not be accurately tested, which underlines that in future studies one needs to control and power for seasonal effects.

As previously mentioned, the majority of the children (87%) had a predominant Gram-negative NP community profile (>50% Gram-negative bacteria). This is probably due to predominance of Gram-negative Moraxella and Haemophilus species known to reside specifically at this body site [3], [4]. On average, 76% of the overall NP microbiome in children was composed of Gram-negative bacteria, however with a wide range of 9–99% Gram-negative bacteria per sample. In addition, there was a higher contribution of Gram-negative bacteria to microbiota obtained in fall/winter (81%) compared to spring (72%) (Independent samples t-test: p = 0.044). This could potentially explain some of the observed differences between gram-negative ratios at the NP microbiome and at other human microbiota, where seasonal changes in composition have so far not been studied.

Finally, we studied the inter-individual diversity in NP microbiota overall and in relation to season. We observed highly diverse microbiota, with on average 40 OTUs per sample, and a high inter-individual diversity with 20–87 OTUs per individual. With respect to season, there was no significant difference in diversity between fall/winter (average: 38 OTUs, range: 20–77) and spring (average: 43 OTUs, range: 20–87) (independent samples T-test: p = 0.083).

As internal control, we compared conventional culturing results for the potential pathogens *S. pneumoniae* (71% positive), *H. influenzae* (69% positive) and *M. catarrhalis* (88% positive) with sequencing results for the OTUs Streptococcus (OTU 3, OTU 23, OTU 31), *H. influenzae* (OTU 2, OTU 10), and Moraxella (OTU 1, OTU 6) respectively (Figure S1) and found strong correlations between *S. pneumoniae* and Streptococcus OTU 3 and 31 (p<0.0001) but not OTU 23, which is probably another Streptococcus species, and *H. influenzae* and *H. influenzae* OTU 2 and OTU 10 (p<0.0001). For *M. catarrhalis*, we were only able to find a positive correlation between M. catarrhalis and Moraxella OTU 6 with independent samples t-test (p = 0.003) but not with Spearman's Correlation, which may be explained by the low number of Moraxella negative individuals, making a comparison between binary and quantitative data difficult. Also the presence of other Moraxella species with high sequence homology might interfere with a strong correlation between these results.

In conclusion, to our knowledge this is the first report describing in detail the composition of and the variability within the human NP microbiota assessed at the depth of next generation sequencing. In line with other human body habitats, we found a complex, diverse and highly variable microbiota with a relatively limited core microbiome. There is considerable seasonal variation in NP microbiota. This implies the time of sampling should be considered when describing or comparing NP microbiomes, and preferably controlled for when other potential determinants like the impact of viruses or antibiotics on microbiota profiles or the correlation between microbiota profiles and diseases will be tested. Whether these seasonal changes in composition of the NP microbome are causally related to seasonal occurrence of respiratory tract infections remains to be determined, though seems relevant for further understanding of pathogenesis of infectious diseases and in the long run potentially for understanding of effects of current and design of future preventive measures.

## Methods

### Samples

We randomly selected 150 NP samples from a cohort of 330 healthy children 18 months of age who had participated in a randomised controlled trial studying the effect of reduced-dose schedules of 7-valent pneumococcal conjugate vaccine (PCV-7) performed in a general community in the Western part of The Netherlands where the control children received PCV-7 only after the trial was finished at the age of 24 months [21]. An acknowledged national ethics committee from the Netherlands (Stichting Therapeutische Evaluatie Geneesmiddelen, http://www.stegmetc.org) approved the study protocol. The trial was undertaken in accordance with the European Statements for Good Clinical Practice, which includes the provisions of the Declaration of Helsinki of 1989. Written informed consent was provided by the parents or their legal guardians.

Nasopharyngeal swabs (Transwab Pernasal Plain (Catalogue MW173P), Medical Wire & Equipment Co, Ltd, Corsham, Wiltshire, England) were collected between November 2007 and June 2008 during home visits after written informed consent was provided by study participants and/or their legal guardians. The swabs were obtained by approaching the nasopharynx transnasally, transported to the laboratory in Transwab (modified Amies) medium (room temperature) and plated within 24 hours on selective agar media. After plating, the cotton swabs were consecutively rinsed in 1 ml of saline and stored at −80°C until further analysis.

### Microbial cultivation

The nasopharyngeal swabs were plated onto a 5% sheep blood agar plate, a 5% sheep blood agar plate with 5 mg/L gentamicin, a chocolate agar plate and a Haemophilus chocolate agar plate. Agar plates were incubated at 35°C for 48 h; the blood agar plate aerobically, the blood agar plate with gentamicin and the chocolate agar plates with raised CO2. Identification of *S. pneumoniae, H. influenzae*, *M. catarrhalis* and *S. aureus* was based on colony morphology and conventional methods of determination.

### Bacterial DNA isolation

One 200 µl aliquot of swab “rinse” solution was distributed in two separate sterile screw-cap Eppendorf tubes, each containing 0.25 ml lysis buffer (AGOWA mag Mini DNA Isolation Kit, catalgue 40410, AGOWA, Berlin, Germany). Then 0.3 g zirconium beads (diameter, 0.1 mm, catalogue 11079101z, Biospec Products, Bartlesville, OK 74005. USA) and 0.2 ml phenol (Phenol solution BioUltra, TE-saturated, catalogue P4557, Sigma-Aldrich, St. Louis, MO, USA) were added to each sample. The samples were homogenized with a Mini-beadbeater (Mini-beadbeater 16, catalogue 607EUR, Biospec Products, Bartlesville, OK 74005. USA) for 2 min. The released DNA was purified with the AGOWA mag Mini DNA Isolation Kit according to the manufacturer's recommendations. To maximize recovery, the DNA binding step was performed twice for each sample. Following, the DNA for each sample was eluted in a total volume of 40 µl milliQ. The integrity of the DNA was inspected by agar gel electrophoresis. DNA was quantified on the NanoDrop spectrophotometer (Thermo Scientific NanoDrop 1000 Spectrophotometer, Thermo Scienific, Wilmington, DE 19810 USA).

### Real time PCR for bacterial DNA

The total bacterial load of the samples was established by quantitative PCR. The primer-probe set targeting the bacterial 16S rDNA gene comprised of forward primer 16S-F1 (5′-CGA AAG CGT GGG GAG CAA A -3′), reverse primer 16S-R1 (5′-GTT CGT ACT CCC CAG GCG G-3′) and probe 16S-P1 (FAM- ATT AGA TAC CCT GGT AGT CCA –MGB). The PCR mixture consisted of 15 µl of 2x master mix (Universal Mastermix,catalogue GMO-UN-A100, Europe Diagenode sa, Liège, Belgium), 1 µl of each primer (10 µM), 1 µl of the probe (5 µM), 9.5 µl DNA free water and 2.5 µl of template DNA. Amplifications were performed using a 7500 Fast Real-Time PCR System (Applied Biosystems, catalogue 4351107, Foster City, CA 94404 USA) under the following conditions: 2 min at 50°C and 10 min at 95°C, followed by 45 cycles of 15 s at 95°C and 1 min at 60°C.

### Amplicon libraries

To generate the PCR amplicon libraries, the small subunit ribosomal RNA gene V5–V6 hypervariable region was amplified for each individual sample independently. Of the 150 samples tested, 96 samples contained more than 1,3*10∧3 fg/µl DNA and were included in sequence analysis. PCR was performed using the forward primer 785F (5′-GGA TTA GAT ACC CBR GTA GTC-3′) and the reverse primer 1061R (5′-TCA CGR CAC GAG CTG ACG AC-3′). The primers were fitted with the 454 Life Sciences Adapter A (forward primer) and B (reverse primer), fused to the 5′ end of the 16S rDNA bacterial primer sequences. The reverse primer also included a unique tetranucleotide sample identification key. The amplification mix contained 2 units of Pfu Ultra II Fusion HS DNA polymerase (Stratagene, La Jolla, CA, USA) and 1x *PfuUltra* II reaction buffer (Stratagene), 200 µM dNTP PurePeak DNA polymerase Mix(Pierce Nucleic Acid Technologies, catalogue NU606001 Milwaukee, WI, USA), and 0.2 µM of each primer. After denaturation (94°C; 2 min), 30 cycles were performed that consisted of denaturation (94°C; 30 sec), annealing (50°C; 40 sec), and extension (72°C; 80 sec). DNA was isolated by means of the MinElute kit (Qiagen, catalogue 28006, Hilden, Germany). The quality and the size of the amplicons were analyzed on the Agilent 2100 Bioanalyser with the DNA 1000 Chip kit (Agilent Technologies, catalogue 5067–1504, Santa Clara, CA, USA) and quantified using Nanodrop ND-1000 spectrophotometer. The amplicons of the individual samples were pooled in equimolar amounts in four libraries. The four libraries were sequenced unidirectionally in the reverse direction (B-adaptor) during two 454 Genome Sequencer FLX (GS-FLX, 454 Life Sciences (Roche), Branford, CT 06405 USA) runs. Sequences are available at the Short Read Archive of the National Center for Biotechnology Information (NCBI) [NCBI SRA: 029327.1].

### Real-time PCR for viruses

One 200 µl aliquot of swab “rinse” solution was used to extract viral nucleic acids using the MagNA Pure LC total nucleic acid isolation kit (Roche Diagnostics, catalogue 03 038 505 001, Basel, Switzerland) as described previously [22]. Detection of viral pathogens was performed in parallel, using real-time PCR assays for bocavirus (HBoV), polyomaviruses (WUPyV and KIPyV), respiratory syncytial virus (RSV) A and B, influenzavirus (IV) A and B, para-influenzavirus (PIV) 1–4, human rhinoviruses (HRV), adenoviruses, human coronavirus OC43, NL63, HKU and 229E, and human metapneumovirus (hMPV). Real-time PCR procedures were performed as described previously [22] Briefly, samples were assayed in duplicate in a 25 µl reaction mixture containing 10 µl (c)DNA, 12.5 µl 2 × TaqMan Universal PCR Master Mix (Applied Biosystems, catalogue 4304437, Foster City, CA 94404 USA), 300–900 nmol/l of the forward and reverse primers and 75–200 nmol/l of each of the probes. All samples had been spiked before extraction with an internal control virus (phocine distemper virus [RNA virus] and phocine herpes virus [DNA virus]) to monitor for efficient extraction and amplification.

### Data Analysis

GS-FLX sequencing data were processed as previously described [23]. In brief, we trimmed data by removing primer sequences and low-quality data, sequences that did not have an exact match to the reverse primer, that had an ambiguous base call (N) in the sequence, or that were shorter than 50 nt after trimming. We then used the GAST algorithm [24] to calculate the percent difference between each unique sequence and its closest match in a database of 69816 unique Eubacterial and 2779 unique Archaeal V5–V6 sequences, representing 323499 SSU rRNA sequences from the SILVA database [25]. Taxa were assigned to each full-length reference sequence using several sources including Entrez Genome entries, cultured strain identities, SILVA, and the Ribosomal Database Project Classifier [26]. In cases where reads were equidistant to multiple V5–V6 reference sequences, and/or where identical V5–V6 sequences were derived from longer sequences mapping to different taxa, reads were assigned to the lowest common taxon of at least two-thirds of the sequences. The operational taxonomic units (OTUs) were created by aligning unique sequences and calculating distance matrices as previously described [23] and using DOTUR [27] to create clusters at the 3% level.

Only those sequences that were found at least 5 times were included in the analyses. This strict and conservative approach was chosen to preclude inclusion of sequences from potential contamination or sequencing artefacts. To compare the relative abundance of OTUs among samples, the data were normalized for number of sequenced reads obtained for each sample. To reduce the influence of abundant taxa on principal component analyses, the normalized abundance data were log2 transformed. Unsupervised data analysis, Principle Component Analysis, and hierarchical clustering was performed using MeV software package as part of TM4 microarray software suite [28].

Seasonal differences in phyla distribution were studied by using Mann Whitney U test (SPSS software Version 15.0). Seasonal differences in OTU patterns as well as potential correlations between respiratory viruses and OTU patterns were studied by the Significant Analysis of Microarrays (SAM analysis) - a non-parametric statistical technique for finding significant differences between microarray data of groups based on experimental conditions [29], implemented in the MeV software package [28]. To determine significant differences between microbiota profiles, we used Pearson's correlation with average linkage clustering method and a FDR significance criterion of <0.05.

Independent samples T-test, and Spearman correlation coefficients (SPSS software Version 15.0) were used for testing correlations between conventional cultures and pyrosequencing data. Independent samples t-test was also used to compare the contribution of gram negative and positive bacteria to the microbiota in different seasons and to test for differences in diversity between seasons.

## Supporting Information

Figure S1 Graphs showing correlation between conventional culture results for *S. pneumoniae, H. influenzae*, and *M. catarrhalis* on the x-axis (absent/present) and sequencing results (as % of total microbiota profile) for Streptococcus (OTU 3), *H. influenzae* (OTU 2 plus 10), and Moraxella (OTU 6) on the y-axis.
(TIFF)

Table S1 Population characteristics for all samples and subdivided per season.
(DOC)

Table S2 Full list of and relative abundance of taxa in the 96 study samples. Here all 243 taxa (species or more inclusive taxa when sequences could not be confidently classified to species level) and their absolute presence per child (yes/no) and relative abundance (% of all sequences) in NP microbiomes are listed. NA- not assigned.
(DOC)

Table S3 Results from q-PCR for detection of respiratory viruses in the 96 nasopharyngeal samples.
(DOC)
