# Supplementary figures and images for: Variability and Diversity of Nasopharyngeal Microbiota in Children: A Metagenomic Analysis
Source: PLoS One. 2011 Feb 28;6(2):e17035. doi: 10.1371/journal.pone.0017035 (PMC3046172; doi:10.1371/journal.pone.0017035)

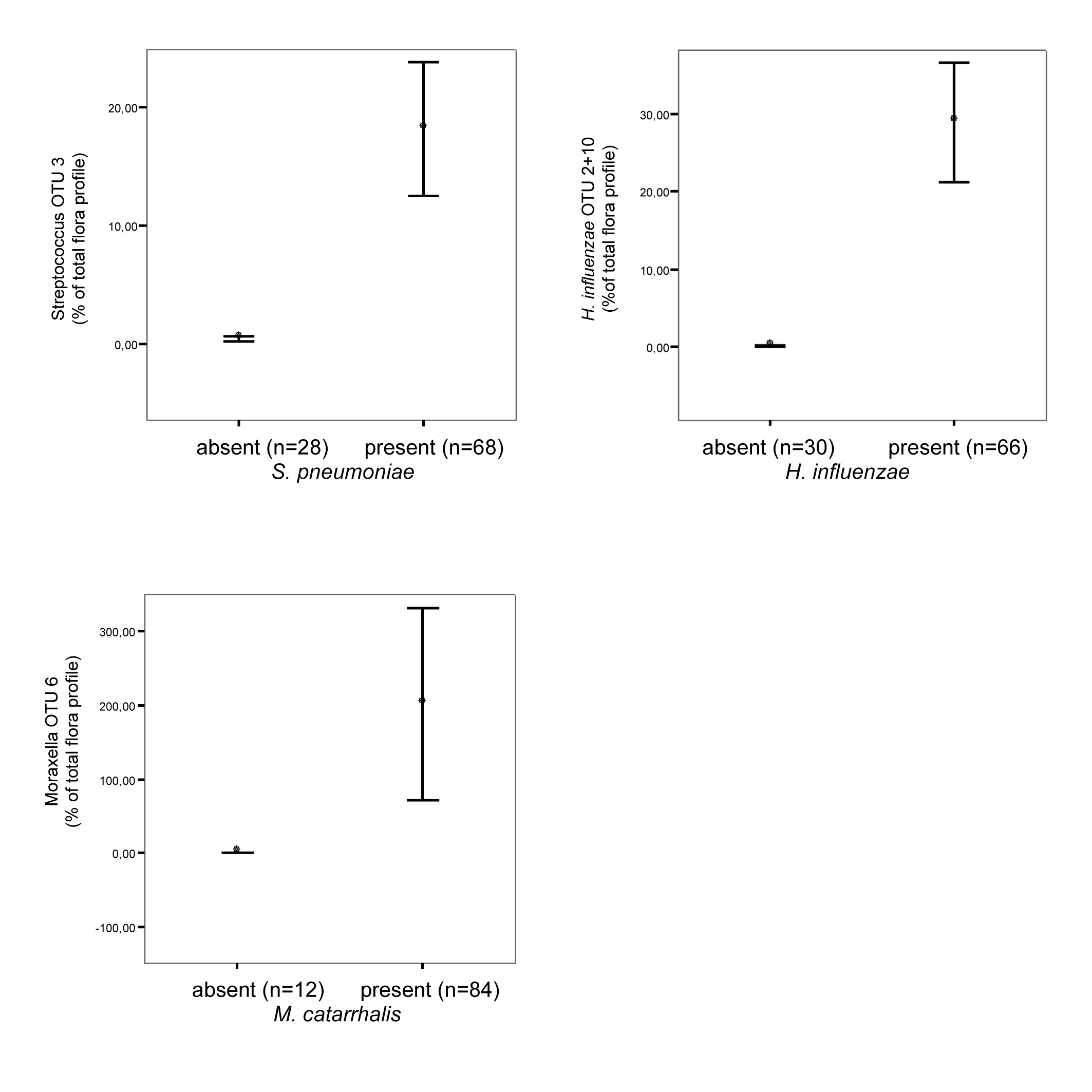

Supplement: Figure S1 — Graphs showing correlation between conventional culture results for S. pneumoniae, H. influenzae, and M. catarrhalis on the x-axis (absent/present) and sequencing results (as % of total microbiota profile) for Streptococcus (OTU 3), H. influenzae (OTU 2 plus 10), and Moraxella (OTU 6) on the y-axis. (TIFF) [file pone.0017035.s001.tiff]
