# Supplementary material for: Variability and Diversity of Nasopharyngeal Microbiota in Children: A Metagenomic Analysis
Source: PLoS One. 2011 Feb 28;6(2):e17035. doi: 10.1371/journal.pone.0017035 (PMC3046172; doi:10.1371/journal.pone.0017035)
Supplement: Table S1 — Population characteristics for all samples and subdivided per season. (DOC) [file pone.0017035.s002.doc]

|  | **Number** | **Range** | **Fall/Winter** | **Spring** | **p-value (Chi^2)^** |
| --- | --- | --- | --- | --- | --- |
| Total population | 96 |  | 50 | 46 |  |
| Age (months) | 18 | 17-19 | 18 (17.5-19) | 18(17.5-19) | NS |
| Gender (boy) | 47 |  | 24 | 23 | NS |
| Pacifier use | 56 |  | 29 | 27 | NS |
| Adenoidectomy | 2 |  | 1 | 1 | NS |
| Tonsillectomy | 0 |  | 0 | 0 | NS |
| Ear ventilation tubes | 2 |  | 2 | 0 | NS |
|  |  |  |  |  |  |
| Smoke exposure at home (yes/no) | 8 |  | 4 | 4 | NS |
| Day-care attendance (yes/no) | 64 |  | 33 | 31 | NS |
| Sibs < 17 years of age (yes/no) | 56 | 1-5 | 32 | 24 | 0.24 |
|  |  |  |  |  |  |
| Current acute otitis media | 4 |  | 2 | 2 | NS |
| Mild symptoms of current cold | 40 |  | 24 | 16 | 0.19 |
| Recent/current wheezing | 12 |  | 6 | 6 | NS |
| Eczema | 13 |  | 7 | 6 | NS |
|  |  |  |  |  |  |
| Use of bronchodilators | 12 |  | 7 | 5 | NS |
| Use of inhalation steroids | 3 |  | 2 | 1 | NS |
| Previous antibiotic consumption | 20 |  | 10 | 10 | NS |
| # within one month prior | 6 |  | 5 | 1 | 0.11 |
| # within two months prior | 11 |  | 7 | 4 | NS |
| # more than two months prior | 12 |  | 4 | 8 | NS |
|  |  |  |  |  |  |
