# Supplementary material for: Variability and Diversity of Nasopharyngeal Microbiota in Children: A Metagenomic Analysis
Source: PLoS One. 2011 Feb 28;6(2):e17035. doi: 10.1371/journal.pone.0017035 (PMC3046172; doi:10.1371/journal.pone.0017035)
Supplement: Table S2 — Full list of and relative abundance of taxa in the 96 study samples. Here all 243 taxa (species or more inclusive taxa when sequences could not be confidently classified to species level) and their absolute presence per child (yes/no) and relative abundance (% of all sequences) in NP microbiomes are listed. NA- not assigned. (DOC) [file pone.0017035.s003.doc]

|  | **Phylum** | **Class** | **Order** | **Family** | **Genus** | **Species** | **OTU level** | **Overall abundance**  **(% of all reads)** | **Relative presence**  **( n = )** |
| --- | --- | --- | --- | --- | --- | --- | --- | --- | --- |
| 1 | Proteobacteria | Gammaproteobacteria | Pseudomonadales | Moraxellaceae | Moraxella | NA | genus | 38.11 | 95 |
| 2 | Proteobacteria | Gammaproteobacteria | Pasteurellales | Pasteurellaceae | Haemophilus | influenzae | species | 19.16 | 84 |
| 3 | Firmicutes | Bacilli | Lactobacillales | Streptococcaceae | Streptococcus | NA | genus | 12.98 | 96 |
| 4 | Bacteroidetes | Flavobacteria | Flavobacteriales | Flavobacteriaceae | Flavobacterium | NA | genus | 10.07 | 80 |
| 5 | Firmicutes | Bacilli | Lactobacillales | Carnobacteriaceae | Dolosigranulum | NA | genus | 4.80 | 86 |
| 6 | Proteobacteria | Gammaproteobacteria | Pseudomonadales | Moraxellaceae | Moraxella | NA | genus | 2.22 | 39 |
| 7 | Actinobacteria | Actinobacteria | Actinomycetales | Corynebacteriaceae | Corynebacterium | propinquum | species | 1.65 | 80 |
| 8 | Proteobacteria | Betaproteobacteria | Neisseriales | Neisseriaceae | Neisseria | meningitidis | species | 1.19 | 62 |
| 9 | Fusobacteria | Fusobacteria | Fusobacteriales | Fusobacteriaceae | Fusobacterium | necrophorum | species | 0.96 | 8 |
| 10 | Proteobacteria | Gammaproteobacteria | Pasteurellales | Pasteurellaceae | Haemophilus | influenzae | species | 0.77 | 16 |
| 11 | Proteobacteria | Betaproteobacteria | Neisseriales | Neisseriaceae | Neisseria | polysaccharea | species | 0.65 | 16 |
| 12 | Firmicutes | Clostridia | Clostridiales | Peptostreptococcaceae | Helcococcus | NA | genus | 0.57 | 31 |
| 13 | Firmicutes | NA | NA | NA | NA | NA | phylum | 0.57 | 49 |
| 14 | Actinobacteria | Actinobacteria | Actinomycetales | Dermabacteraceae | Brachybacterium | NA | genus | 0.56 | 16 |
| 15 | Fusobacteria | Fusobacteria | Fusobacteriales | Fusobacteriaceae | Fusobacterium | NA | genus | 0.40 | 21 |
| 16 | Proteobacteria | Gammaproteobacteria | Pseudomonadales | Moraxellaceae | Enhydrobacter | NA | genus | 0.37 | 95 |
| 17 | Proteobacteria | Gammaproteobacteria | Pasteurellales | Pasteurellaceae | Haemophilus | NA | genus | 0.34 | 14 |
| 18 | Bacteroidetes | Bacteroidia | Bacteroidales | Porphyromonadaceae | Porphyromonas | catoniae | species | 0.27 | 29 |
| 19 | Firmicutes | Bacilli | Lactobacillales | Lactobacillaceae | Lactobacillus | NA | genus | 0.24 | 17 |
| 20 | Bacteroidetes | Bacteroidia | Bacteroidales | Porphyromonadaceae | Porphyromonas | catoniae | species | 0.21 | 23 |
| 21 | Firmicutes | Clostridia | Clostridiales | Peptostreptococcaceae | Parvimonas | NA | Genus | 0.19 | 8 |
| 22 | Cyanobacteria | NA | NA | NA | NA | NA | phylum | 0.18 | 83 |
| 23 | Firmicutes | Bacilli | Lactobacillales | Streptococcaceae | Streptococcus | NA | Genus | 0.17 | 25 |
| 24 | Firmicutes | Bacilli | Bacillales | Paenibacillaceae | Brevibacillus | brevis | species | 0.16 | 43 |
| 25 | Bacteroidetes | Bacteroidia | Bacteroidales | Prevotellaceae | Prevotella | shahii | species | 0.15 | 1 |
| 26 | Firmicutes | Bacilli | Bacillales | Bacillaceae | Bacillus | NA | Genus | 0.14 | 42 |
| 27 | Actinobacteria | Actinobacteria | Actinomycetales | Propionibacteriaceae | Propionibacterium | NA | Genus | 0.13 | 90 |
| 28 | Firmicutes | Bacilli | Bacillales | Staphylococcaceae | Staphylococcus | NA | Genus | 0.12 | 80 |
| 29 | Firmicutes | Clostridia | Clostridiales | Lachnospiraceae | NA | NA | Family | 0.12 | 15 |
| 30 | Proteobacteria | Betaproteobacteria | Burkholderiales | Comamonadaceae | Acidovorax | NA | Genus | 0.11 | 86 |
| 31 | Firmicutes | Bacilli | Lactobacillales | Streptococcaceae | Streptococcus | NA | Genus | 0.10 | 12 |
| 32 | Proteobacteria | Gammaproteobacteria | Pasteurellales | Pasteurellaceae | NA | NA | Family | 0.09 | 25 |
| 33 | Bacteroidetes | Bacteroidia | Bacteroidales | NA | NA | NA | Orderx | 0.08 | 30 |
| 34 | Firmicutes | Bacilli | Lactobacillales | Carnobacteriaceae | Granulicatella | NA | Genus | 0.08 | 50 |
| 35 | Firmicutes | Bacilli | Lactobacillales | Lactobacillaceae | Lactobacillus | NA | Genus | 0.08 | 10 |
| 36 | Actinobacteria | Actinobacteria | Actinomycetales | Microbacteriaceae | Microbacterium | NA | Genus | 0.08 | 9 |
| 37 | Firmicutes | Bacilli | Bacillales | Bacillaceae | Bacillus | NA | Genus | 0.08 | 45 |
| 38 | Bacteroidetes | Flavobacteria | Flavobacteriales | Flavobacteriaceae | Chryseobacterium | NA | Genus | 0.07 | 2 |
| 39 | Proteobacteria | Gammaproteobacteria | Enterobacteriales | Enterobacteriaceae | NA | NA | Family | 0.06 | 57 |
| 40 | Firmicutes | Bacilli | Lactobacillales | Lactobacillaceae | Lactobacillus | helveticus | species | 0.06 | 11 |
| 41 | Proteobacteria | Betaproteobacteria | Burkholderiales | Burkholderiaceae | Ralstonia | NA | Genus | 0.06 | 51 |
| 42 | Bacteroidetes | Bacteroidia | Bacteroidales | Prevotellaceae | Prevotella | NA | Genus | 0.06 | 5 |
| 43 | Proteobacteria | Betaproteobacteria | Burkholderiales | Oxalobacteraceae | NA | NA | Family | 0.05 | 52 |
| 44 | Proteobacteria | Gammaproteobacteria | Pseudomonadales | Moraxellaceae | Moraxella | NA | Genus | 0.05 | 31 |
| 45 | Firmicutes | Bacilli | Lactobacillales | Streptococcaceae | Streptococcus | NA | Genus | 0.05 | 63 |
| 46 | Actinobacteria | Actinobacteria | NA | NA | NA | NA | Class | 0.05 | 49 |
| 47 | Proteobacteria | Gammaproteobacteria | Pasteurellales | Pasteurellaceae | Haemophilus | influenzae | species | 0.04 | 32 |
| 48 | Proteobacteria | Gammaproteobacteria | Pseudomonadales | Moraxellaceae | Acinetobacter | NA | Genus | 0.04 | 70 |
| 49 | Actinobacteria | Actinobacteria | Actinomycetales | Microbacteriaceae | NA | NA | Family | 0.04 | 42 |
| 50 | Proteobacteria | Betaproteobacteria | Burkholderiales | Oxalobacteraceae | NA | NA | Family | 0.04 | 53 |
| 51 | Firmicutes | Clostridia | Clostridiales | Peptostreptococcaceae | NA | NA | Family | 0.04 | 9 |
| 52 | Proteobacteria | Alphaproteobacteria | Rhizobiales | NA | NA | NA | Orderx | 0.04 | 41 |
| 53 | TM7 | NA | NA | NA | NA | NA | phylum | 0.03 | 8 |
| 54 | Proteobacteria | Gammaproteobacteria | Pseudomonadales | Pseudomonadaceae | Pseudomonas | NA | Genus | 0.03 | 40 |
| 55 | Firmicutes | Clostridia | Clostridiales | Lachnospiraceae | Catonella | NA | Genus | 0.03 | 3 |
| 56 | Proteobacteria | Alphaproteobacteria | NA | NA | NA | NA | Class | 0.03 | 42 |
| 57 | OD1 | NA | NA | NA | NA | NA | phylum | 0.03 | 57 |
| 58 | Actinobacteria | Actinobacteria | Actinomycetales | Microbacteriaceae | NA | NA | Family | 0.03 | 35 |
| 59 | Proteobacteria | Gammaproteobacteria | Pseudomonadales | Moraxellaceae | Moraxella | NA | Genus | 0.03 | 26 |
| 60 | Proteobacteria | Alphaproteobacteria | Caulobacterales | Caulobacteraceae | Brevundimonas | NA | Genus | 0.03 | 15 |
| 61 | Actinobacteria | Actinobacteria | Actinomycetales | Micrococcaceae | Rothia | NA | Genus | 0.03 | 44 |
| 62 | Fusobacteria | Fusobacteria | Fusobacteriales | Leptotrichiaceae | Streptobacillus | moniliformis | species | 0.02 | 11 |
| 63 | Proteobacteria | Alphaproteobacteria | Rhizobiales | Methylobacteriaceae | Methylobacterium | NA | Genus | 0.02 | 8 |
| 64 | OD1 | NA | NA | NA | NA | NA | phylum | 0.02 | 44 |
| 65 | Firmicutes | Clostridia | Clostridiales | Peptococcaceae | Peptococcus | NA | Genus | 0.02 | 3 |
| 66 | Firmicutes | Clostridia | Clostridiales | Veillonellaceae | NA | NA | Family | 0.02 | 38 |
| 67 | Proteobacteria | Gammaproteobacteria | Pseudomonadales | Moraxellaceae | Acinetobacter | NA | genus | 0.02 | 27 |
| 68 | Actinobacteria | Actinobacteria | Actinomycetales | Corynebacteriaceae | Corynebacterium | NA | genus | 0.02 | 34 |
| 69 | Firmicutes | Clostridia | Clostridiales | Veillonellaceae | NA | NA | family | 0.02 | 20 |
| 70 | Actinobacteria | Actinobacteria | Actinomycetales | Micrococcaceae | Micrococcus | NA | genus | 0.02 | 38 |
| 71 | Proteobacteria | Gammaproteobacteria | Pasteurellales | Pasteurellaceae | Haemophilus | NA | genus | 0.02 | 32 |
| 72 | Bacteroidetes | Flavobacteria | Flavobacteriales | Flavobacteriaceae | Cloacibacterium | NA | genus | 0.02 | 9 |
| 73 | Actinobacteria | Actinobacteria | Bifidobacteriales | Bifidobacteriaceae | Bifidobacterium | animalis | species | 0.02 | 26 |
| 74 | Firmicutes | Bacilli | Lactobacillales | Lactobacillaceae | Lactobacillus | NA | genus | 0.02 | 6 |
| 75 | Firmicutes | Bacilli | Bacillales | Bacillaceae | Bacillus | NA | genus | 0.02 | 33 |
| 76 | Proteobacteria | Gammaproteobacteria | Pseudomonadales | Pseudomonadaceae | Pseudomonas | NA | genus | 0.015 | 27 |
| 77 | Firmicutes | Bacilli | Bacillales | Bacillaceae | Halobacillus | NA | genus | 0.015 | 21 |
| 78 | Firmicutes | Bacilli | Lactobacillales | Streptococcaceae | Lactococcus | NA | genus | 0.015 | 25 |
| 79 | Firmicutes | Bacilli | Bacillales | Planococcaceae | Kurthia | NA | genus | 0.014 | 8 |
| 80 | Proteobacteria | Gammaproteobacteria | Pseudomonadales | Moraxellaceae | Psychrobacter | NA | genus | 0.014 | 20 |
| 81 | Actinobacteria | Actinobacteria | Actinomycetales | Corynebacteriaceae | Corynebacterium | NA | genus | 0.013 | 32 |
| 82 | Bacteroidetes | Sphingobacteria | Sphingobacteriales | Sphingobacteriaceae | Sphingobacterium | NA | genus | 0.013 | 1 |
| 83 | Bacteroidetes | Sphingobacteria | Sphingobacteriales | Sphingobacteriaceae | Sphingobacterium | NA | genus | 0.012 | 1 |
| 84 | Bacteroidetes | Bacteroidia | Bacteroidales | Bacteroidaceae | Bacteroides | fragilis | species | 0.012 | 13 |
| 85 | Firmicutes | Bacilli | Lactobacillales | Lactobacillaceae | Lactobacillus | NA | genus | 0.012 | 8 |
| 86 | Bacteroidetes | NA | NA | NA | NA | NA | phylum | 0.012 | 1 |
| 87 | Actinobacteria | Actinobacteria | Actinomycetales | Actinomycetaceae | Actinomyces | NA | genus | 0.012 | 29 |
| 88 | Firmicutes | Clostridia | Clostridiales | Peptostreptococcaceae | Anaerococcus | NA | genus | 0.011 | 12 |
| 89 | Firmicutes | Bacilli | Bacillales | Bacillaceae | Anoxybacillus | NA | genus | 0.011 | 23 |
| 90 | Actinobacteria | Actinobacteria | Actinomycetales | Brevibacteriaceae | NA | NA | family | 0.011 | 20 |
| 91 | Bacteroidetes | Flavobacteria | Flavobacteriales | Flavobacteriaceae | Flavobacterium | NA | genus | 0.011 | 1 |
| 92 | Proteobacteria | Deltaproteobacteria | Myxococcales | Polyangiaceae | Byssovorax | NA | genus | 0.010 | 11 |
| 93 | Actinobacteria | Actinobacteria | Actinomycetales | Micrococcaceae | Arthrobacter | NA | genus | 0.010 | 6 |
| 94 | Proteobacteria | Gammaproteobacteria | Enterobacteriales | Enterobacteriaceae | Klebsiella | NA | genus | 0.010 | 17 |
| 95 | Actinobacteria | Actinobacteria | Actinomycetales | Micrococcaceae | Kocuria | NA | genus | 0.010 | 9 |
| 96 | Deinococcus-Thermus | Deinococci | Thermales | Thermaceae | Thermus | NA | genus | 0.008 | 26 |
| 97 | Actinobacteria | Actinobacteria | Actinomycetales | Microbacteriaceae | Leucobacter | NA | genus | 0.007 | 1 |
| 98 | Actinobacteria | Actinobacteria | Actinomycetales | NA | NA | NA | orderx | 0.007 | 22 |
| 99 | Actinobacteria | Actinobacteria | Actinomycetales | Actinomycetaceae | Arcanobacterium | hippocoleae | species | 0.007 | 14 |
| 100 | OD1 | NA | NA | NA | NA | NA | phylum | 0.007 | 24 |
| 101 | Bacteroidetes | Bacteroidia | Bacteroidales | Prevotellaceae | Prevotella | NA | genus | 0.006 | 2 |
| 102 | Firmicutes | Clostridia | Clostridiales | Ruminococcaceae | NA | NA | family | 0.006 | 3 |
| 103 | Proteobacteria | Alphaproteobacteria | Rhodobacterales | Rhodobacteraceae | Paracoccus | NA | genus | 0.006 | 1 |
| 104 | Proteobacteria | Betaproteobacteria | Burkholderiales | Oxalobacteraceae | NA | NA | family | 0.006 | 15 |
| 105 | Proteobacteria | Alphaproteobacteria | Rhizobiales | Phyllobacteriaceae | Mesorhizobium | NA | genus | 0.006 | 20 |
| 106 | Firmicutes | Bacilli | Bacillales | Listeriaceae | Listeria | NA | genus | 0.006 | 19 |
| 107 | Proteobacteria | Gammaproteobacteria | Alteromonadales | Shewanellaceae | Shewanella | NA | genus | 0.006 | 10 |
| 108 | Bacteroidetes | Bacteroidia | Bacteroidales | Prevotellaceae | Prevotella | nanceiensis | species | 0.006 | 3 |
| 109 | Proteobacteria | Gammaproteobacteria | Pasteurellales | Pasteurellaceae | Pasteurella | NA | genus | 0.005 | 9 |
| 110 | Proteobacteria | Gammaproteobacteria | Aeromonadales | Aeromonadaceae | Aeromonas | NA | genus | 0.005 | 11 |
| 111 | Bacteria | NA | NA | NA | NA | NA | domain | 0.005 | 8 |
| 112 | Proteobacteria | Gammaproteobacteria | Aeromonadales | Aeromonadaceae | Aeromonas | NA | genus | 0.005 | 9 |
| 113 | Proteobacteria | Alphaproteobacteria | Rhizobiales | Methylocystaceae | Methylocystis | NA | genus | 0.005 | 15 |
| 114 | Firmicutes | Clostridia | Clostridiales | Clostridiaceae | Clostridium | NA | genus | 0.005 | 8 |
| 115 | Firmicutes | Clostridia | Clostridiales | Lachnospiraceae | NA | NA | family | 0.005 | 8 |
| 116 | Actinobacteria | Actinobacteria | Actinomycetales | Nocardiaceae | Rhodococcus | NA | genus | 0.005 | 8 |
| 117 | Actinobacteria | Actinobacteria | Actinomycetales | Actinomycetaceae | Actinomyces | NA | genus | 0.005 | 15 |
| 118 | Actinobacteria | Actinobacteria | Actinomycetales | Micrococcaceae | Rothia | NA | genus | 0.004 | 11 |
| 119 | Proteobacteria | Betaproteobacteria | Neisseriales | Neisseriaceae | NA | NA | family | 0.004 | 13 |
| 120 | Proteobacteria | Betaproteobacteria | Rhodocyclales | Rhodocyclaceae | Propionivibrio | NA | genus | 0.004 | 13 |
| 121 | Bacteroidetes | Bacteroidia | Bacteroidales | Rikenellaceae | Alistipes | NA | genus | 0.004 | 6 |
| 122 | Firmicutes | Clostridia | Clostridiales | Ruminococcaceae | Faecalibacterium | NA | genus | 0.004 | 4 |
| 123 | Firmicutes | Clostridia | Clostridiales | Lachnospiraceae | NA | NA | family | 0.004 | 1 |
| 124 | Firmicutes | Bacilli | Lactobacillales | Aerococcaceae | Abiotrophia | NA | genus | 0.004 | 6 |
| 125 | Actinobacteria | Actinobacteria | Actinomycetales | Sanguibacteraceae | Sanguibacter | NA | genus | 0.004 | 6 |
| 126 | Bacteroidetes | Sphingobacteria | Sphingobacteriales | NA | NA | NA | orderx | 0.004 | 14 |
| 127 | Firmicutes | Bacilli | Bacillales | Paenibacillaceae | Paenibacillus | NA | genus | 0.004 | 11 |
| 128 | Deinococcus-Thermus | Deinococci | Thermales | Thermaceae | Thermus | NA | genus | 0.004 | 13 |
| 129 | Actinobacteria | Actinobacteria | Actinomycetales | Corynebacteriaceae | Corynebacterium | kroppenstedtii | species | 0.004 | 10 |
| 130 | Actinobacteria | Actinobacteria | Actinomycetales | Intrasporangiaceae | NA | NA | family | 0.004 | 4 |
| 131 | Nitrospira | NA | NA | NA | NA | NA | phylum | 0.004 | 13 |
| 132 | Firmicutes | Bacilli | Bacillales | Bacillaceae | Geobacillus | NA | genus | 0.003 | 13 |
| 133 | Firmicutes | Clostridia | Clostridiales | Ruminococcaceae | NA | NA | family | 0.003 | 7 |
| 134 | Proteobacteria | Betaproteobacteria | Burkholderiales | Alcaligenaceae | Derxia | NA | genus | 0.003 | 13 |
| 135 | Proteobacteria | Alphaproteobacteria | Rhizobiales | Bradyrhizobiaceae | Bradyrhizobium | NA | genus | 0.003 | 14 |
| 136 | Proteobacteria | Betaproteobacteria | Neisseriales | Neisseriaceae | NA | NA | family | 0.003 | 1 |
| 137 | Proteobacteria | Betaproteobacteria | NA | NA | NA | NA | class | 0.003 | 15 |
| 138 | Actinobacteria | Actinobacteria | Actinomycetales | Corynebacteriaceae | Corynebacterium | NA | genus | 0.003 | 11 |
| 139 | Firmicutes | Clostridia | Clostridiales | Peptostreptococcaceae | Peptoniphilus | NA | genus | 0.003 | 7 |
| 140 | Firmicutes | Clostridia | Clostridiales | Clostridiaceae | Clostridium | baratii | species | 0.003 | 5 |
| 141 | Proteobacteria | Gammaproteobacteria | Xanthomonadales | Sinobacteraceae | Nevskia | NA | genus | 0.003 | 17 |
| 142 | Bacteroidetes | Bacteroidia | Bacteroidales | Bacteroidaceae | Bacteroides | NA | genus | 0.003 | 5 |
| 143 | Bacteroidetes | Sphingobacteria | Sphingobacteriales | NA | NA | NA | orderx | 0.003 | 10 |
| 144 | Proteobacteria | Alphaproteobacteria | Rhodobacterales | Rhodobacteraceae | Paracoccus | NA | genus | 0.003 | 10 |
| 145 | Proteobacteria | Alphaproteobacteria | Rhodobacterales | Rhodobacteraceae | Paracoccus | NA | genus | 0.003 | 4 |
| 146 | Actinobacteria | Actinobacteria | Actinomycetales | Micrococcaceae | Kocuria | NA | genus | 0.003 | 4 |
| 147 | Proteobacteria | Betaproteobacteria | Burkholderiales | Comamonadaceae | Comamonas | NA | genus | 0.002 | 1 |
| 148 | Bacteroidetes | Bacteroidia | Bacteroidales | Prevotellaceae | Prevotella | NA | genus | 0.002 | 10 |
| 149 | Proteobacteria | Betaproteobacteria | Neisseriales | Neisseriaceae | Kingella | kingae | species | 0.002 | 1 |
| 150 | Proteobacteria | Betaproteobacteria | NA | NA | NA | NA | class | 0.002 | 10 |
| 151 | Actinobacteria | Actinobacteria | Actinomycetales | Pseudonocardiaceae | Pseudonocardia | NA | genus | 0.002 | 2 |
| 152 | Firmicutes | Erysipelotrichi | Erysipelotrichales | Erysipelotrichaceae | Coprobacillus | NA | genus | 0.002 | 4 |
| 153 | Firmicutes | Clostridia | Clostridiales | Ruminococcaceae | Papillibacter | NA | genus | 0.002 | 1 |
| 154 | Proteobacteria | Betaproteobacteria | Burkholderiales | Comamonadaceae | Delftia | NA | genus | 0.002 | 1 |
| 155 | Actinobacteria | Actinobacteria | Coriobacteriales | Coriobacteriaceae | Collinsella | NA | genus | 0.002 | 13 |
| 156 | Actinobacteria | Actinobacteria | Actinomycetales | Corynebacteriaceae | Corynebacterium | NA | genus | 0.002 | 4 |
| 157 | Firmicutes | Clostridia | Clostridiales | Veillonellaceae | Selenomonas | NA | genus | 0.002 | 1 |
| 158 | Firmicutes | Bacilli | Lactobacillales | Lactobacillaceae | Lactobacillus | gallinarum | species | 0.002 | 1 |
| 159 | Actinobacteria | Actinobacteria | Actinomycetales | Nocardiaceae | Rhodococcus | NA | genus | 0.002 | 4 |
| 160 | Firmicutes | Clostridia | Clostridiales | Ruminococcaceae | Subdoligranulum | NA | genus | 0.002 | 7 |
| 161 | OD1 | NA | NA | NA | NA | NA | phylum | 0.002 | 9 |
| 162 | Proteobacteria | Betaproteobacteria | Rhodocyclales | Rhodocyclaceae | Dechloromonas | NA | genus | 0.002 | 7 |
| 163 | Planctomycetes | NA | NA | NA | NA | NA | phylum | 0.002 | 4 |
| 164 | Firmicutes | NA | NA | NA | NA | NA | phylum | 0.002 | 1 |
| 165 | Bacteroidetes | Bacteroidia | Bacteroidales | Prevotellaceae | Prevotella | NA | genus | 0.002 | 1 |
| 166 | Firmicutes | Clostridia | Clostridiales | Lachnospiraceae | NA | NA | family | 0.002 | 3 |
| 167 | Firmicutes | Clostridia | Clostridiales | Peptostreptococcaceae | NA | NA | family | 0.002 | 3 |
| 168 | Proteobacteria | Alphaproteobacteria | Rhodobacterales | Rhodobacteraceae | Paracoccus | NA | genus | 0.002 | 5 |
| 169 | Proteobacteria | Betaproteobacteria | Burkholderiales | NA | NA | NA | orderx | 0.002 | 6 |
| 170 | Firmicutes | Bacilli | Lactobacillales | Carnobacteriaceae | Dolosigranulum | NA | genus | 0.002 | 10 |
| 171 | Firmicutes | Clostridia | Clostridiales | Lachnospiraceae | NA | NA | family | 0.002 | 2 |
| 172 | Proteobacteria | Betaproteobacteria | Burkholderiales | Burkholderiaceae | Burkholderia | NA | genus | 0.002 | 4 |
| 173 | Bacteroidetes | Sphingobacteria | Sphingobacteriales | Sphingobacteriaceae | Sphingobacterium | spiritivorum | species | 0.001 | 1 |
| 174 | Firmicutes | Bacilli | Lactobacillales | Streptococcaceae | Streptococcus | NA | genus | 0.001 | 8 |
| 175 | Firmicutes | Clostridia | Clostridiales | Lachnospiraceae | NA | NA | family | 0.001 | 4 |
| 176 | Actinobacteria | Actinobacteria | Actinomycetales | Corynebacteriaceae | Corynebacterium | NA | genus | 0.001 | 4 |
| 177 | Firmicutes | Clostridia | Clostridiales | Ruminococcaceae | NA | NA | family | 0.001 | 2 |
| 178 | Proteobacteria | Gammaproteobacteria | Xanthomonadales | Xanthomonadaceae | Stenotrophomonas | NA | genus | 0.001 | 5 |
| 179 | Firmicutes | Bacilli | Lactobacillales | Streptococcaceae | Streptococcus | cristatus | species | 0.001 | 4 |
| 180 | Actinobacteria | Actinobacteria | Bifidobacteriales | Unassigned | Turicella | NA | genus | 0.001 | 6 |
| 181 | Firmicutes | Clostridia | Clostridiales | Lachnospiraceae | NA | NA | family | 0.001 | 1 |
| 182 | Proteobacteria | Gammaproteobacteria | Pseudomonadales | Moraxellaceae | Acinetobacter | NA | genus | 0.001 | 6 |
| 183 | Proteobacteria | Gammaproteobacteria | Xanthomonadales | Xanthomonadaceae | Rhodanobacter | NA | genus | 0.001 | 4 |
| 184 | Bacteroidetes | Flavobacteria | Flavobacteriales | Flavobacteriaceae | Capnocytophaga | sputigena | species | 0.001 | 3 |
| 185 | Actinobacteria | Actinobacteria | Bifidobacteriales | Bifidobacteriaceae | Bifidobacterium | NA | genus | 0.001 | 6 |
| 186 | Fusobacteria | Fusobacteria | Fusobacteriales | Leptotrichiaceae | Leptotrichia | NA | genus | 0.001 | 2 |
| 187 | Bacteroidetes | Bacteroidia | Bacteroidales | Bacteroidaceae | Bacteroides | NA | genus | 0.001 | 3 |
| 188 | Proteobacteria | Gammaproteobacteria | NA | NA | NA | NA | class | 0.001 | 6 |
| 189 | Actinobacteria | Actinobacteria | Actinomycetales | Gordoniaceae | Gordonia | NA | genus | 0.001 | 1 |
| 190 | Firmicutes | Clostridia | Clostridiales | Lachnospiraceae | NA | NA | family | 0.001 | 1 |
| 191 | Actinobacteria | Actinobacteria | Actinomycetales | Corynebacteriaceae | Corynebacterium | NA | genus | 0.001 | 5 |
| 192 | Proteobacteria | Betaproteobacteria | Burkholderiales | Comamonadaceae | Pelomonas | NA | genus | 0.001 | 4 |
| 193 | Bacteroidetes | Flavobacteria | Flavobacteriales | Flavobacteriaceae | Capnocytophaga | NA | genus | 0.001 | 1 |
| 194 | Actinobacteria | Actinobacteria | Actinomycetales | Brevibacteriaceae | Brevibacterium | NA | genus | 0.001 | 2 |
| 195 | Actinobacteria | Actinobacteria | Actinomycetales | Propionibacteriaceae | Propionibacterium | NA | genus | 0.001 | 1 |
| 196 | Proteobacteria | Betaproteobacteria | Burkholderiales | Oxalobacteraceae | Massilia | NA | genus | 0.001 | 2 |
| 197 | Bacteroidetes | Flavobacteria | Flavobacteriales | Flavobacteriaceae | Cloacibacterium | NA | genus | 0.001 | 4 |
| 198 | Proteobacteria | Alphaproteobacteria | Rhizobiales | Brucellaceae | Ochrobactrum | NA | genus | 0.001 | 1 |
| 199 | Actinobacteria | Actinobacteria | Actinomycetales | Corynebacteriaceae | Corynebacterium | durum | species | 0.001 | 2 |
| 200 | Proteobacteria | NA | NA | NA | NA | NA | phylum | 0.001 | 4 |
| 201 | Firmicutes | Bacilli | Bacillales | Paenibacillaceae | Paenibacillus | NA | genus | 0.001 | 6 |
| 202 | Firmicutes | Clostridia | Clostridiales | Peptostreptococcaceae | Anaerococcus | octavius | species | 0.001 | 6 |
| 203 | Proteobacteria | Gammaproteobacteria | Aeromonadales | Aeromonadaceae | Aeromonas | NA | genus | 0.001 | 1 |
| 204 | Firmicutes | Clostridia | Clostridiales | Lachnospiraceae | NA | NA | family | 0.001 | 1 |
| 205 | Firmicutes | Clostridia | Clostridiales | Lachnospiraceae | NA | NA | family | 0.001 | 1 |
| 206 | Chloroflexi | NA | NA | NA | NA | NA | phylum | 0.001 | 4 |
| 207 | Proteobacteria | Gammaproteobacteria | Pasteurellales | Pasteurellaceae | Haemophilus | NA | genus | 0.001 | 2 |
| 208 | Actinobacteria | Actinobacteria | Bifidobacteriales | Bifidobacteriaceae | Bifidobacterium | NA | genus | 0.001 | 3 |
| 209 | Fusobacteria | Fusobacteria | Fusobacteriales | Leptotrichiaceae | Leptotrichia | NA | genus | 0.001 | 3 |
| 210 | Firmicutes | Clostridia | Clostridiales | Clostridiaceae | Clostridium | NA | genus | 0.001 | 1 |
| 211 | Proteobacteria | Gammaproteobacteria | Pseudomonadales | Moraxellaceae | NA | NA | family | 0.001 | 3 |
| 212 | Firmicutes | Erysipelotrichi | Erysipelotrichales | Erysipelotrichaceae | NA | NA | family | 0.001 | 1 |
| 213 | Firmicutes | Bacilli | Lactobacillales | Lactobacillaceae | Lactobacillus | NA | genus | 0.001 | 1 |
| 214 | Firmicutes | Clostridia | Clostridiales | Lachnospiraceae | NA | NA | family | 0.001 | 1 |
| 215 | Firmicutes | Clostridia | Clostridiales | Peptostreptococcaceae | NA | NA | family | 0.001 | 1 |
| 216 | Firmicutes | Clostridia | Clostridiales | Peptostreptococcaceae | NA | NA | family | 0.001 | 1 |
| 217 | Proteobacteria | Betaproteobacteria | Neisseriales | Neisseriaceae | Chitinilyticum | aquatile | species | 0.001 | 2 |
| 218 | Proteobacteria | Alphaproteobacteria | Rhizobiales | NA | NA | NA | orderx | 0.001 | 7 |
| 219 | Proteobacteria | Alphaproteobacteria | Rhizobiales | Bradyrhizobiaceae | Bradyrhizobium | NA | genus | 0.001 | 5 |
| 220 | Proteobacteria | Alphaproteobacteria | Rhodospirillales | Acetobacteraceae | Rubritepida | flocculans | species | 0.001 | 1 |
| 221 | Firmicutes | Bacilli | Lactobacillales | Lactobacillaceae | Pediococcus | NA | genus | 0.001 | 1 |
| 222 | Proteobacteria | Alphaproteobacteria | NA | NA | NA | NA | class | 0.001 | 2 |
| 223 | Firmicutes | Clostridia | Clostridiales | Ruminococcaceae | Ruminococcus | NA | genus | 0.001 | 1 |
| 224 | Firmicutes | Clostridia | Clostridiales | Peptostreptococcaceae | NA | NA | family | 0.001 | 1 |
| 225 | Firmicutes | Clostridia | Clostridiales | Lachnospiraceae | NA | NA | family | 0.001 | 1 |
| 226 | Firmicutes | Clostridia | Clostridiales | Lachnospiraceae | NA | NA | family | 0.001 | 1 |
| 227 | Firmicutes | Clostridia | Clostridiales | Lachnospiraceae | NA | NA | family | 0.001 | 1 |
| 228 | Firmicutes | Bacilli | Bacillales | Bacillaceae | Bacillus | NA | genus | 0.001 | 1 |
| 229 | Firmicutes | Clostridia | Clostridiales | Lachnospiraceae | NA | NA | family | 0.001 | 1 |
| 230 | Proteobacteria | Alphaproteobacteria | Sphingomonadales | Sphingomonadaceae | Sphingomonas | NA | genus | 0.001 | 3 |
| 231 | Firmicutes | Clostridia | Clostridiales | NA | NA | NA | orderx | 0.001 | 3 |
| 232 | Firmicutes | Clostridia | Clostridiales | Lachnospiraceae | NA | NA | family | 0.001 | 4 |
| 233 | Proteobacteria | Gammaproteobacteria | Xanthomonadales | Xanthomonadaceae | Pseudoxanthomonas | NA | genus | 0.001 | 3 |
| 234 | Proteobacteria | Alphaproteobacteria | Sphingomonadales | Sphingomonadaceae | Novosphingobium | NA | genus | 0.001 | 2 |
| 235 | Firmicutes | Clostridia | Clostridiales | Lachnospiraceae | NA | NA | family | 0.001 | 1 |
| 236 | BRC1 | NA | NA | NA | NA | NA | phylum | 0.001 | 1 |
| 237 | Proteobacteria | Betaproteobacteria | Burkholderiales | Unassigned | Thiobacter | NA | genus | 0.001 | 3 |
| 238 | Proteobacteria | Alphaproteobacteria | Rhizobiales | Rhizobiaceae | Rhizobium | NA | genus | 0.001 | 1 |
| 239 | Actinobacteria | Actinobacteria | NA | NA | NA | NA | class | 0.001 | 3 |
| 240 | Proteobacteria | Gammaproteobacteria | Pseudomonadales | Moraxellaceae | Psychrobacter | NA | genus | 0.0005 | 4 |
| 241 | Actinobacteria | Actinobacteria | Actinomycetales | Corynebacteriaceae | Corynebacterium | propinquum | species | 0.0005 | 4 |
| 242 | OD1 | NA | NA | NA | NA | NA | phylum | 0.0003 | 1 |
| 243 | Proteobacteria | Deltaproteobacteria | NA | NA | NA | NA | class | 0.0003 | 1 |
