# Supplementary material for: Variability and Diversity of Nasopharyngeal Microbiota in Children: A Metagenomic Analysis
Source: PLoS One. 2011 Feb 28;6(2):e17035. doi: 10.1371/journal.pone.0017035 (PMC3046172; doi:10.1371/journal.pone.0017035)
Supplement: Table S3 — Results from q-PCR for detection of respiratory viruses in the 96 nasopharyngeal samples. (DOC) [file pone.0017035.s004.doc]

| **Virus present:** | **% all** | **% in Fall/Winter** | **% in Spring** |
| --- | --- | --- | --- |
| Human rhinovirus | 44 | 34 | 54 |
| Bocavirus | 21 | 18 | 24 |
| Polyomaviruses | 14 | 14 | 13 |
| Adenovirus | 11 | 8 | 15 |
| Para-influenzavirus I_IV | 11 | 10 | 13 |
| Coronavirus | 10 | 20 | 0 |
| Human metapneumovirus | 0 | 0 | 0 |
| Influenza A_B virus | 1 | 2 | 0 |
| Respiratory syncytial virus A_B | 1 | 2 | 0 |
|  |  |  |  |
| Any virus | 67 | 64 | 70 |
| - Single virus | 31 | 26 | 35 |
| - Two or more viruses | 36 | 38 | 35 |
